# Synthesis of Vertically Aligned ZnO Nanorods Using Sol-gel Seeding and Colloidal Lithography Patterning

**DOI:** 10.1186/s11671-021-03500-7

**Published:** 2021-03-12

**Authors:** Ebrahim Chalangar, Omer Nur, Magnus Willander, Anders Gustafsson, Håkan Pettersson

**Affiliations:** 1 Department of Science and Technology, Physics, Electronics and Mathematics, Linköping University, Norrköping, Sweden; 2 School of Information Technology, Halmstad University, 301 18 Halmstad, Sweden; 3 Solid State Physics and NanoLund, Lund University, Box 118, 221 00 Lund, Sweden

**Keywords:** Zinc oxide, Nanorod arrays, Vertical growth, Colloidal lithography, Nanofabrication, Sol-gel, Seed layer, Chemical bath deposition

## Abstract

Different ZnO nanostructures can be grown using low-cost chemical bath deposition. Although this technique is cost-efficient and flexible, the final structures are usually randomly oriented and hardly controllable in terms of homogeneity and surface density. In this work, we use colloidal lithography to pattern (100) silicon substrates to fully control the nanorods' morphology and density. Moreover, a sol-gel prepared ZnO seed layer was employed to compensate for the lattice mismatch between the silicon substrate and ZnO nanorods. The results show a successful growth of vertically aligned ZnO nanorods with controllable diameter and density in the designated openings in the patterned resist mask deposited on the seed layer. Our method can be used to fabricate optimized devices where vertically ordered ZnO nanorods of high crystalline quality are crucial for the device performance.

**Supplementary Information:**

The online version contains supplementary material available at 10.1186/s11671-021-03500-7.

## Introduction

The ability to realize a desirable precise nanomaterial architecture with respect to dimensions, arrangement, and density of the nanostructures is crucial for most advanced nanodevices. One viable route to accomplish this is to combine a top–bottom patterning technique with a self-assembling bottom-up growth method.

Zinc oxide (ZnO), one of the most widely studied semiconductors, shows an excellent potential for self-assembled optoelectronic nanodevices. Among the key properties of ZnO in this context can be mentioned, a simple growth procedure, a wide direct bandgap of 3.2–3.4 eV, a large exciton binding energy of 60 meV and a high mechanical and thermal stability [1]. Different high-temperature growth techniques such as chemical vapor deposition (CVD) [2], pulsed laser deposition (PLD) [3] and vapor–liquid–solid (VLS) growth [4], as well as low-temperature growth techniques including electrodeposition [5] and chemical bath deposition (CBD) [6] have been used to grow a plenitude of ZnO nanostructures. Among all these growth techniques, CBD is more favorable due to its simplicity, cost-effectiveness and large-area applicability.

One-dimensional (1D) wurtzite ZnO nanorods (ZnO NRs) have attracted huge interest in the last two decades due to their interesting fundamental electronic and mechanical properties, as well as to their great promise for novel electronics [6], photonics [7], electrochemical [8] and clean-tech applications. The growth of ZnO NRs using CBD is highly dependent on the crystallographic surface quality of the substrate. The two most frequently used substrates to this date are i) single crystal lattice-matched substrates (single-crystalline ZnO [9], Al_2_O_3_ [10] and GaN [11]) with a thin heteroepitaxial ZnO film, and ii) non-epitaxial substrates precoated with a textured seed layer to provide suitable nucleation sites [12]. While single-crystalline substrates generally result in significantly higher NR growth quality, the high substrate cost limits their application. Conversely, the growth of NRs on low-cost non-epitaxial substrates, supplied with a textured polycrystalline seed layer, leads to a randomly oriented NR growth.

In many high-performance device applications, a precise engineering of the NR surface density, lateral ordering, and vertical alignment is of great importance. Growth of NRs by CBD offers several straightforward ways to engineer the NR architecture on the substrate by controlling growth parameters such as solution concentration [13], temperature [14], pH [15], and deposition duration. Unfortunately, changing any of these growth parameters typically also causes undesired changes in the NR growth result. For this reason, up to now, substrate patterning is the most effective way to control the position, density and alignment of ZnO NRs. In the vast majority of reported studies, patterning of the substrate includes the following steps: substrate preparation and cleaning, deposition of a mask resist layer, opening holes in the resist layer by lithography for selective area growth, and CBD growth of NRs in the holes.

During the past decade, various efforts have been made to grow ordered vertical ZnO NR arrays. In the majority of these studies, using the same CBD technique, the authors have focused on two key processing steps: preparation of appropriate substrates, and usage of different patterning techniques, e.g., electron-beam lithography (EBL), laser interference lithography (LIL), and nanoimprint lithography (NIL). Wang et al. demonstrated non-epitaxial growth of vertically aligned ZnO NRs on EBL-patterned polycrystalline ZnO-coated Si and GaN substrates [11]. Later, they reported on perfectly aligned heteroepitaxial ZnO NR growth on GaN substrates patterned by LIL [16]. In an effort to replace the expensive ZnO and GaN substrates with low-cost Si or glass substrates, they successfully used a 30-µm-thick textured ZnO layer as a flat (0001) ZnO seed layer. In a series of articles, Volk et al. successfully performed homoepitaxial growth of ZnO NRs on single-crystalline ZnO substrates patterned by EBL [10, 17–19]. The impact of different patterned substrates [10], a Zn-terminated versus O-terminated surface of the ZnO substrate [17], a sputtered polycrystalline ZnO thin film [18], and a ZnO seed layer deposited by atomic layer deposition (ALD) [19] on the quality of CBD-grown ZnO NRs have been deeply investigated. In a different approach, PLD was used for heteroepitaxial growth of a ZnO seed layer on a single-crystalline Al_2_O_3_ (0001) substrate, followed by NIL patterning [20]. More details about the Zn concentration window in CBD and its effect on the ZnO NR morphology were discussed in the same article. Selective area growth of ZnO NRs on different substrates, including Si (111), GaAs (111) and InP (111), using EBL patterning and electrochemical deposition has also been reported [21].

In all the reviewed references, the growth of ZnO NRs on a single-crystalline ZnO substrate results in the highest crystal quality, while a coating with a thin polycrystalline ZnO layer on other expensive single-crystalline substrates leads to excellent vertically aligned ZnO NR growth. Employing sophisticated patterning techniques, e.g., EBL, for selective growth of ZnO NRs also has an essential impact on the final product cost and, consequently, on the potential application. To overcome demanding complexity issues related to pattern definition and expensive substrates in the fabrication of vertically aligned ZnO NR arrays, we propose to combine a simply prepared, highly uniform thin ZnO NP film on Si substrates with colloidal lithography (CL) patterning.

A sol-gel technique, described in [22], can be used to prepare a highly uniform, dense and thin seed layer of polycrystalline ZnO NPs on virtually any substrate, including Si or glass. In contrast to the spin-coating of a ZnO NP dispersion on a substrate, which usually leads to non-continuous dispersed islands of NP clusters [23], the sol-gel method results in a thin (tens of nm) continuous and highly uniform layer of ZnO NPs. For selective area CBD growth, a polymer resist layer is subsequently spin-coated on the ZnO seed layer and patterned by CL. Besides being a low-cost lithography method, CL is also suitable for large wafer-scale area patterning, high throughput, and compatibility with any substrate, offering excellent control over feature size and pitch [24–26].

**Fig. 1:**
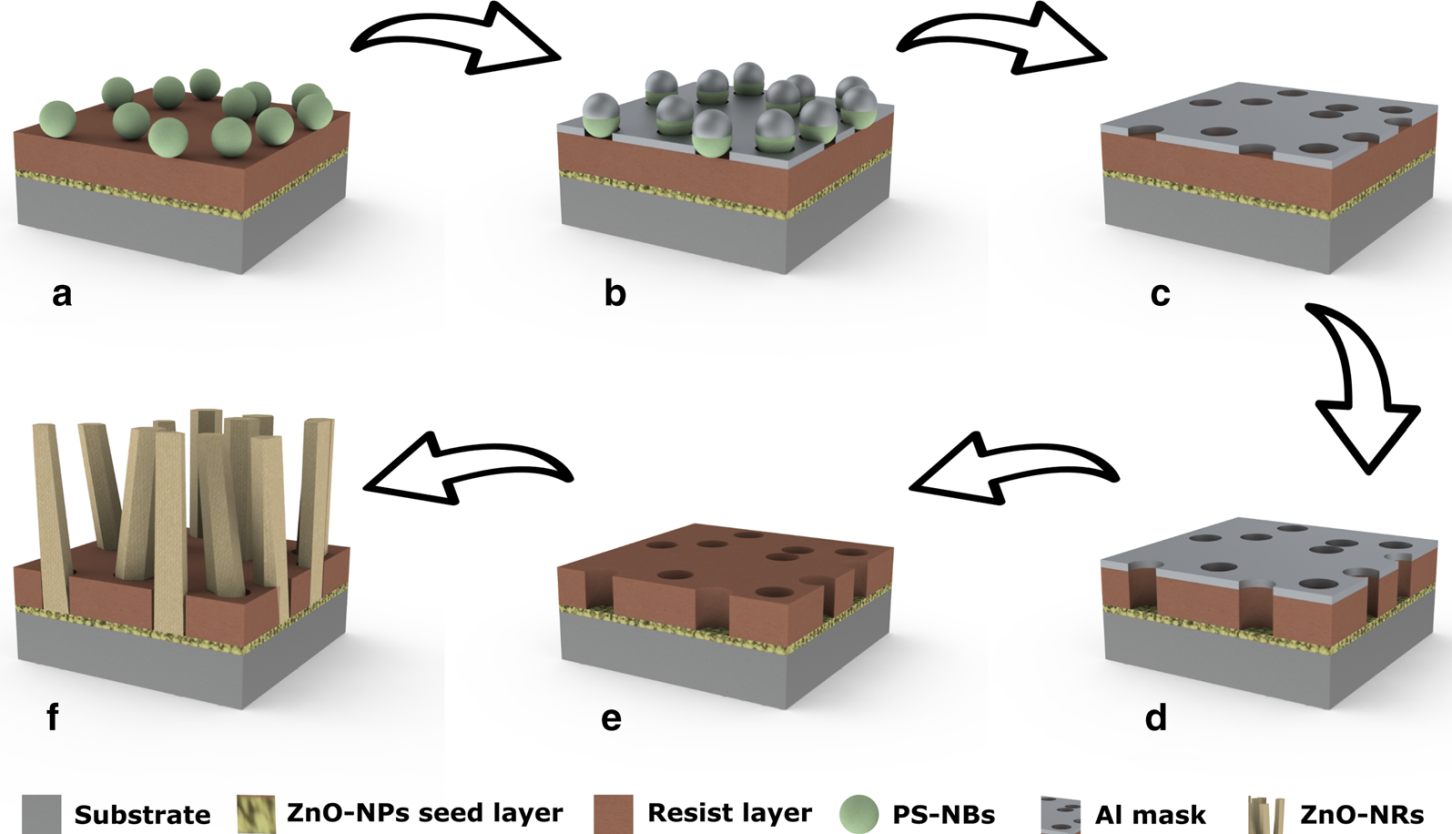
Schematic of the fabrication process steps to synthesize vertically aligned ZnO NR arrays. **a** A substrate coated with a ZnO NP seed layer, a polymer resist layer and PS-NBs. **b** Thermal evaporation of an Al mask. **c** Removing the Al-coated PS-NBs by tape stripping. **d** Dry etching of the resist. **e** Wet etching of the Al mask. **f** Selective ZnO NR growth in the holes of the patterned resist template

Figure 1 shows a schematic of the processing and growth steps used in the present work. After dip-coating the Si substrate with a ZnO NP seed layer and spin-coating with a resist, respectively, a monolayer of polystyrene nanobeads (PS-NBs) with a diameter of 140 nm was deposited on the resist mask and covered by a thermally evaporated 30 nm thick Al film. After evaporation, the Al-coated PS-NBs were removed by tape stripping, leaving behind open nanoholes in the Al film. Dry etching of the resist in the holes down to the ZnO NP seed layer finalized the preparation of the growth mask. After CL, CBD was used to grow vertically aligned ZnO NRs with well-controlled diameter, length and surface density. While CL and CBD have previously been deployed to grow ZnO NRs on micrometer-sized areas on ITO glass substrates, resulting in large uncontrolled batches of grown NRs [27], the present study is, to the best of our knowledge, the first demonstrated selective growth of single vertically aligned ZnO NRs in CL-patterned masks on sol-gel prepared Si substrates. The results show a uniform distribution of nanoholes on a large wafer-scale area and a homogeneous growth of vertical ZnO NRs using a facile and controllable CBD technique.

## Methods

### Materials

All the chemicals used in this work were purchased from Sigma-Aldrich and used without any further purification. The suspension of PS-NBs with a mean diameter of 140 ± 5 nm in water was purchased from microParticles GmbH, Germany.

### Preparation of the ZnO NP Seed Layer

The ZnO NP sol-gel solution was prepared according to [22] by dissolving 8.25 g of zinc acetate (Zn(CH_3_COO)_2_·2H_2_O) and 2.26 ml of monoethanolamine (ethanolamine) in 100 ml of pure ethanol with final concentrations of 375 mM. The solution was stirred on a hotplate at 60 °C for 10 h and then at room temperature overnight. A two-inch, highly doped n-type Si (100) wafer was cleaned by sequential ultrasonication in acetone, isopropanol, and DI water, followed by drying on a hotplate at 120 °C for 5 min. The cleaned Si substrate was dipped into the ZnO NP sol-gel solution and pulled out at a constant speed of 30 mm/s using a computer-controlled dip-coater. Finally, it was annealed on a hotplate at 300 °C for 10 min to remove the residual organics and improve the ZnO NP crystallinity. The dip-coating and the annealing steps were repeated two times to increase the layer homogeneity and surface coverage.

### Patterning the Seeded Sample by CL

The CL was carried out on a double-layer resist, consisting of a sacrificial PMMA (poly(methyl methacrylate), *M*_*W*_ = 950 k) electron-beam resist and a positive MICROPOSIT S1805 photoresist. First, 0.3 ml of a 4 wt% solution of PMMA in toluene was spin coated on the seeded sample at 4000 rpm for 30 s, followed by soft baking at 170 °C for 10 min. Subsequently, 0.3 ml of S1805 was spin coated on the sample at 4000 rpm for 30 s, followed by soft baking at 110 °C for 90 s and hard baking at 145 °C for 5 min. Next, the sample was treated by UV-ozone for 5 min, improving the surface hydrophilicity, and then 1 ml of a 0.2 wt% solution of PDDA (poly(diallyldimethylammonium), *M*_*W*_ = (200–350)k) in DI water was pipetted on the sample surface. After 1 min. of settling, the sample was thoroughly rinsed with DI water and blow-dried with N_2_. The positively charged monolayer of PDDA guarantees electrostatic adhesion of negatively charged PS-NBs to the surface in the next step. Afterward, 1 ml of a 0.1 wt% suspension of PS-NBs in DI water was dropped on the sample surface. After 1 min. of settling, the sample was gently rinsed with DI water, and blow-dried with N_2_. A 30 nm thick Al layer was thermally evaporated on the beaded sample as a metal etch mask. After evaporation, the Al-coated PS-NBs were removed by tape stripping using acrylic silicon-free Ultron 1009R tape (Fig. 1c). Reactive-ion etching (RIE, pressure 150 mTorr, O_2_ flow 40 sccm, RF power 60 W and etching time 90 s) was employed to etch the resist layer down to the ZnO NP seed layer (Fig. 1d). Finally, to wet-etch the Al metal mask (Fig. 1e) without etching the exposed ZnO NP seed layer, a particular solution of potassium hydroxide (KOH) and potassium hexacyanoferrate III (K_3_Fe(CN)_6_) in DI water with a concentration of 30 mM and 50 mM respectively was used [28]. The wet etching was conducted at room temperature for 1 min. Scanning electron microscopy (SEM) and atomic force microscopy (AFM) images of the fabrication steps are provided in Additional file 1: Figures S1 and S2.

### CBD of ZnO NRs on the CL-Patterned Sample

A solution of 50 mM zinc nitrate hexahydrate (Zn(NO_3_)_2_·6H_2_O) and 50 mM hexamethylenetetramine (HMT) in 100 ml DI water was prepared to grow the ZnO NRs. The patterned seeded substrates were kept upside down in the growth solution for 2 h at 95 °C in an oven. After the growth was completed, the samples were cooled to room temperature, removed from the solution, and rinsed with DI water (Fig. 1f).

## Results and Discussion

### ZnO Seed Layer

**Fig. 2:**
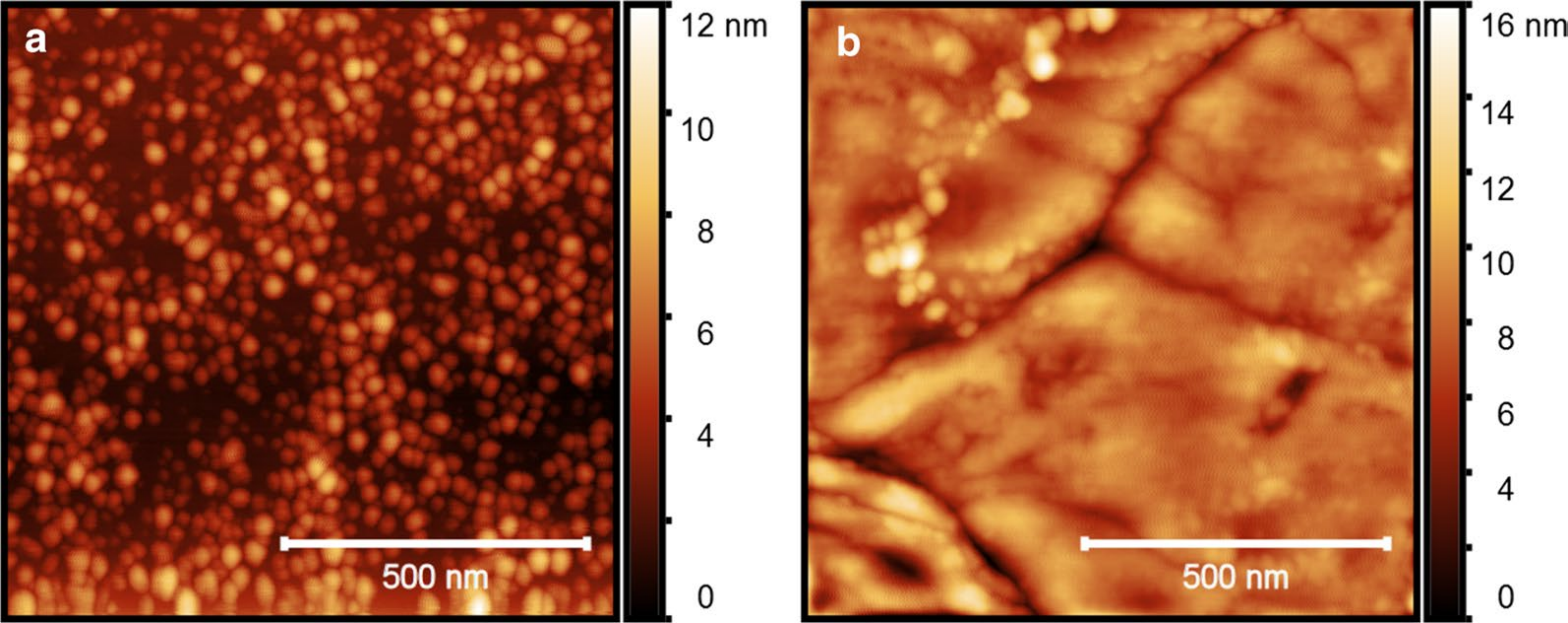
AFM images of ZnO NP seed layers prepared on top of a Si wafer by a sol-gel method after **a** one- and **b** two-step dip-coatings, respectively, followed by an annealing step at 300 °C after each coating. A polynomial background has been subtracted from the AFM images

The growth of well-aligned vertical ZnO NRs with high crystallinity relies on the quality of the seed layer. For this, we developed smooth seed layers with large grain sizes and optimal crystal orientation. The prepared ZnO NP seed layer was investigated with respect to surface roughness and crystal structure using AFM and X-ray diffraction (XRD) analysis. The AFM images in Fig. 2 show significant improvement in the homogeneity and the smoothness of the seed layer after repeating the dip-coating, as explained above. After a single-step dip-coating of a Si substrate in the sol-gel solution, a 21 ± 5 nm thick ZnO NP layer with an RMS roughness of 1.2 nm (Fig. 2a) was formed on the Si surface. Repeating the dip-coating resulted in a smoother 40 ± 5 nm thick ZnO NP layer with an RMS roughness of 0.9 nm at a grain boundary (Fig. 2b). Each dip-coating step was followed by an annealing treatment at 300 °C for 10 min that sintered the NPs together into bigger crystal grain sizes.

Three factors mainly influence the crystal grain boundaries observed in AFM images: (1) the thickness of the ZnO seed layer that is proportional to the number of dip-coating steps, (2) the temperature and the duration of subsequent annealing steps, and (3) impurities or dopants present in the crystal structure. It has been shown that larger grain sizes are achieved by increasing the thickness of the ZnO layers [29, 30]. Also, annealing at a higher temperature and for a longer time leads to better sintering of the small particles and increased grain sizes. In addition, unintentional impurities present during the synthesis process, or added dopants to the ZnO seed layer results in smaller grain sizes [31]. Although our samples are not intentionally doped, the presence of monoethanolamine in the sol-gel solution most likely introduces impurities during the annealing, causing smaller grain sizes. The crystal grain zones are comparably larger than the 140 nm diameter PS-NBs used for the CL. Consequently, the probability of ending up with grown ZnO NRs in holes on top of a single crystal grain without a grain boundary is increased.

**Fig. 3:**
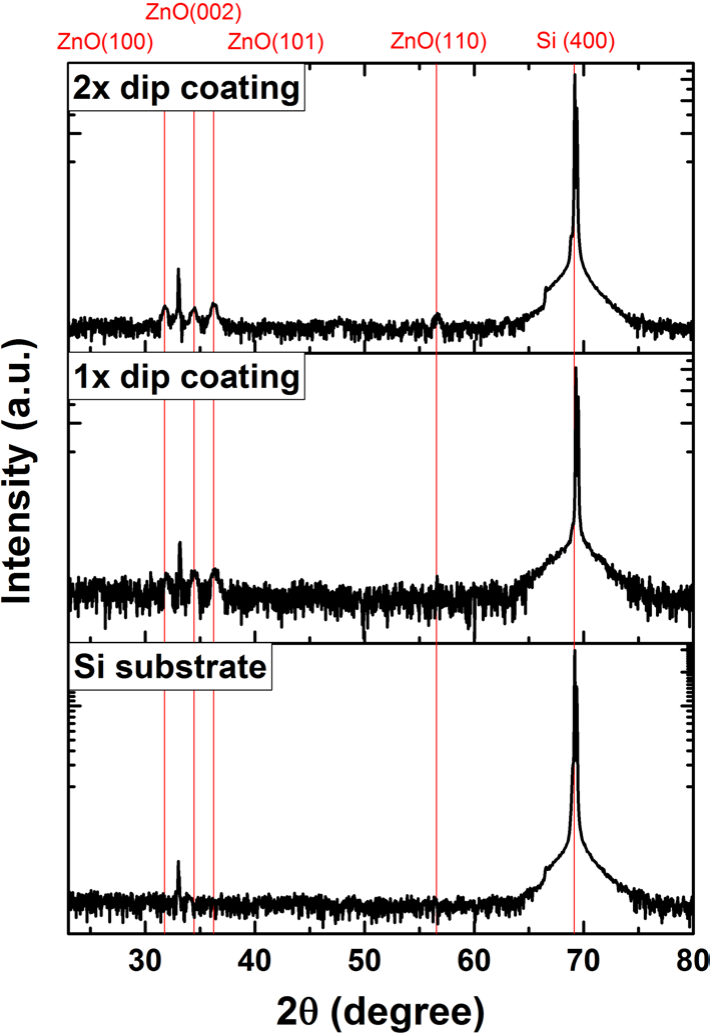
XRD diffraction pattern of a bare Si substrate and of ZnO NP seed layers deposited on a (100) Si substrate by dip-coating. Each dip-coating is followed by an annealing step at 300 °C for 10 min

To grow vertically aligned ZnO NRs, a (002)-oriented ZnO seed layer is desirable. The XRD results in Fig. 3 show the crystal structure and orientation of one- and two-step dip-coated seed layers. In both samples, the polycrystalline ZnO seed layer exhibits crystal plane orientations of (100), (002) and (101). The self-forming process of polycrystalline ZnO NPs has previously been studied in more detail [5, 32]. Applying the second dip-coating and annealing treatment increased the ratio between the integrated XRD peak areas produced by the (100) and (002) planes in agreement with a previous report [29]. Although repeating the dip-coating process improves the seed layer smoothness and grain size, the crystal structure thus becomes less favorable for ZnO NR growth by the increased presence of (100) planes.

### CL patterning and ZnO NRs Growth

**Fig. 4:**
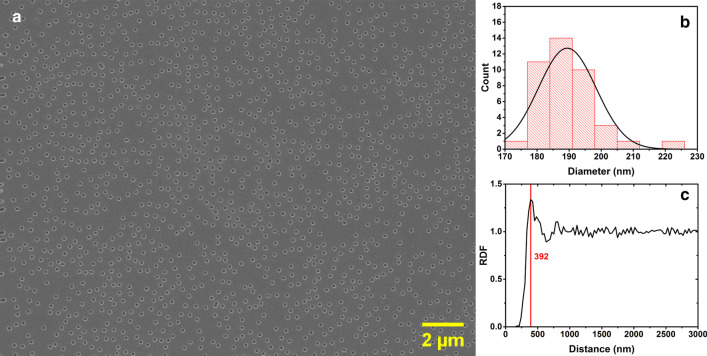
**a** SEM image of a CL-patterned resist layer using 140-nm-diameter PS-NBs on a ZnO NP-seeded Si substrate, after RIE etching and Al wet etching. **b** Distribution of the diameter of the etched nanoholes in the resist layer. **c** The radial distribution function of the patterned nanoholes, with an average nanohole neighboring separation of 392 nm

The deposition of the ZnO seed layers was directly followed by CL-patterning of selected areas. Figure 4a shows an SEM image of a CL-patterned resist layer after RIE etching and Al removal. A uniform, large-area CL patterning is clearly demonstrated on the ZnO-seeded Si substrate, with a nanohole surface density of 4.2 nanohole/µm^2^ and a diameter distribution centered around 190 nm (Fig. 4b). The final nanohole diameter was found to be 36% larger than the diameter of the PS-NBs, which is attributed to the 90 s RIE of the resist layer. The diameter of the nanoholes can simply be tuned by choosing a different PS-NB size. Here, we chose an optimized 140 nm bead size to ensure a single ZnO NR growth in each nanohole by CBD. Using a smaller PS-NB size makes the CBD growth more difficult due to the insufficient flow of the growth solution into the hydrophobic patterned resist. Using larger diameter PS-NBs instead results in undesired growth of clusters of ZnO NRs in each nanohole [21, 27, 33].

In addition to the nanohole feature size, the pitch of the pattern can also be tuned by controlling the average distance between the dispersed PS-NBs. Negatively charged PS-NBs are distributed on the surface by electrostatic net repulsive forces, which can be controlled by screening the electrostatic repulsion forces between the PS-NBs. This has been done by adding a controlled amount of salt into the PS-NB suspension, as previously reported at length in literature [25, 26]. Here we instead considered the effect of PS-NB concentration on the nanohole surface distribution. In addition to the 0.1 wt% PS-NB suspension mentioned above, three lower concentrations of 0.02 wt%, 0.01 wt% and 0.003 wt% were used to fabricate CL-patterned samples. Additional file 1: Figure S4 shows that a reduction of the NB concentration to the mentioned values results in nanohole surface densities of 3.2, 1.5, and 0.4 nanohole/µm^2^, respectively. Interestingly, the radial distribution function (RDF) of the nanoholes for the different samples (Fig. 4c and Additional file 1: S4d) shows a short-range order in the distribution of the nanoholes due to the net force between the PS-NBs in the CL process. From the extracted primary peak positions for different PS-NBs concentrations, it was extracted that the average nanohole separation to its neighbor counterintuitively reduces with decreasing PS-NB concentration to 392 nm, 374 nm, 336 nm, and 298 nm, respectively. From this can be concluded that lower PS-NB concentrations result in a less uniform nanohole distribution, as visible in the SEM images in Additional file 1: Figure S4.

**Fig. 5:**
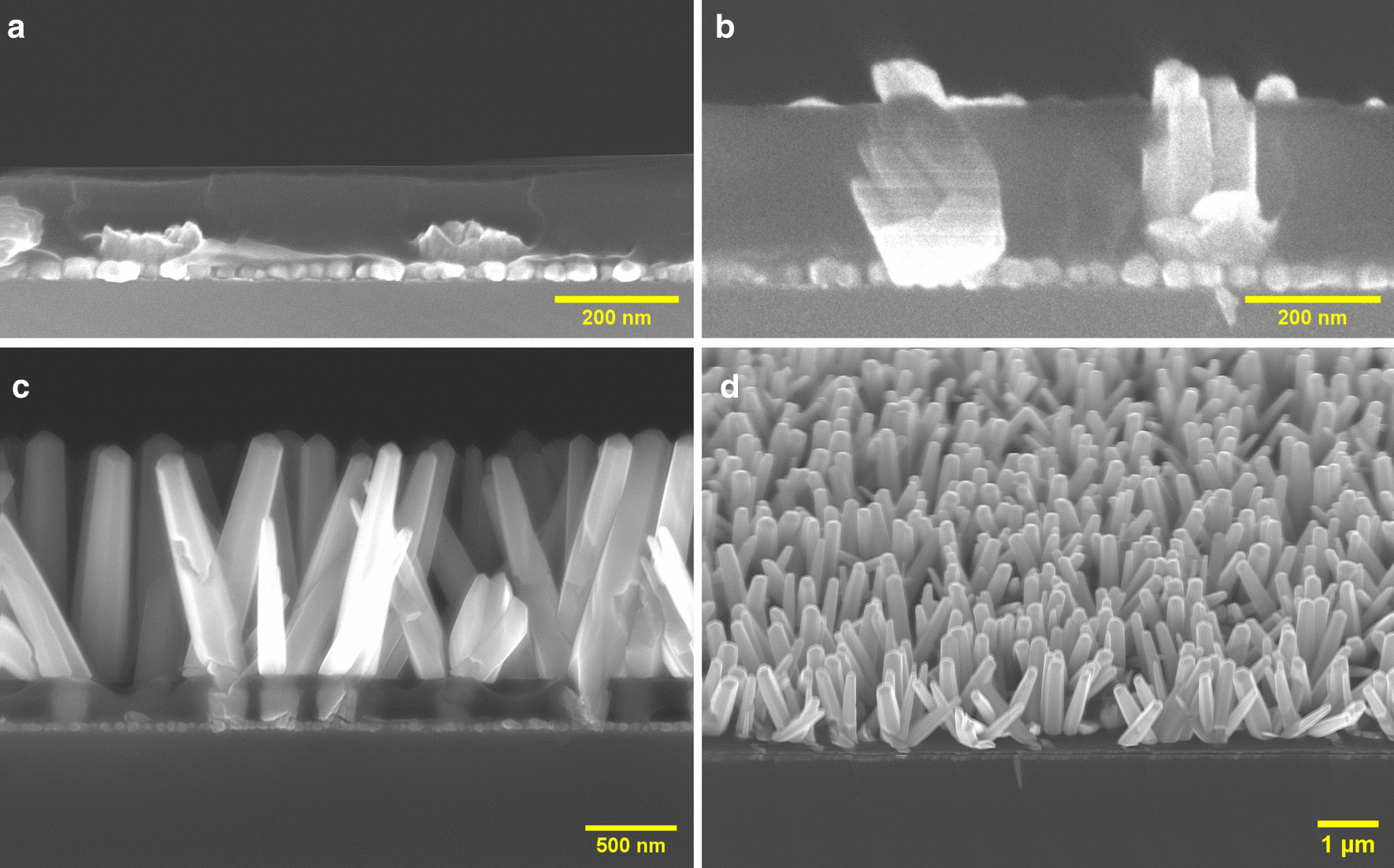
Cross-sectional SEM images of CBD-grown ZnO NRs on CL-patterned ZnO NP seed layers on (100) Si substrates after a growth time of **a** 5 min, **b** 25 min, **c** 2 h and **d** 2 h at 45° tilt

To investigate the growth mechanism of ZnO NRs on the CL-patterned substrates more in detail, a study of the growth rate versus growth time was carried out. Figure 5a, and 5b show the early stages of the ZnO NR growth after 5 min and 25 min, respectively. In the beginning, the exposed opened seeded holes form a wetting layer template for the growth solution with random crystal orientation, providing nucleation sites for CBD of ZnO NRs. Multiple ZnO NRs epitaxially grow from the nuclei sites along random directions (Fig. 5a). The nanohole walls restrict the growth of NRs with large deviation angles, and only those few NRs with a near-vertical direction will continue to grow, as seen in Fig. 5b.

By continuing the CBD process, the nanoholes are filled up by short off-direction NR stubs and only a few NRs grow out from the patterned resist layer. At a high enough growth temperature (95 °C in our experiment), a few near-vertical ZnO NRs in each nanohole merge together, forming a single NR growing out from each opening in agreement with a previous report [11]. The optimal ZnO NR array obtained on a large wafer-scale sample is shown in Fig. 5c and 5d.

**Fig. 6:**
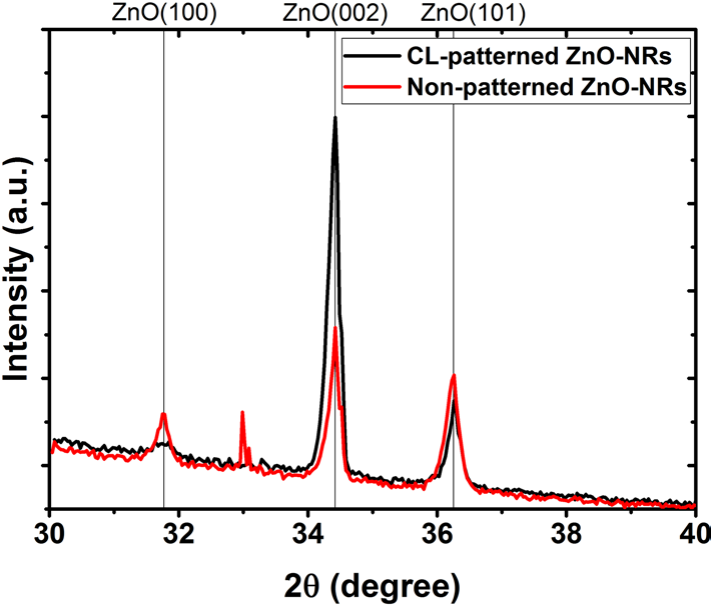
XRD diffraction pattern of CBD-grown ZnO NRs on similar non-patterned and CL-patterned seed layers

To quantify the vertical NR alignment, we performed an XRD analysis of ZnO NR growth on similar non-patterned and CL-patterned seed layers. Figure 6 shows that the ZnO (002) reflection is dominant for the CL-patterned sample, indicating a better ZnO NR c-axis alignment. In contrast, the non-patterned sample shows more pronounced ZnO (100) and (101) reflections resulting from a poor vertical alignment. In addition, a statistical analysis of the SEM images of CL-patterned (Fig. 5c) and non-patterned samples (Additional file 1: Figure S3a) was carried out. The distributions of the deviation angle from the surface normal in Additional file 1: Figures S3b and S3c show a mean deviation angle of 18° for the non-patterned sample and 13° for the CL-patterned sample, respectively. This result provides further support for the conclusion that ZnO NR growth on CL-patterned samples yields better vertical alignment compared to non-patterned samples.

Two additional CL-patterned samples, using 107-nm- and 320-nm-diameter PS-NBs, were prepared and examined by SEM (images added in Additional file 1: Figure S5). The small 107-nm-diameter PS-NBs resulted in a poor and inhomogeneous CBD of ZnO NRs while the bigger 320-nm-diameter PS-NBs led to rather uniform, but multiple star-shaped and randomly aligned ZnO NRs. This result strongly indicates that choosing an optimal nanohole size, depending on the diameter of the NRs, is crucial to grow a single, vertically aligned ZnO NR in each nanohole.

A chemical analysis of the final optimal CBD-grown ZnO NR array was done using dispersive X-ray spectroscopy (EDS). The spatial EDS maps (Additional file 1: Figure S6) show clear presence of the expected elements O, Zn, Si and C. The thin ZnO seed layer was not identified due to the spatial resolution limit.

**Fig. 7:**
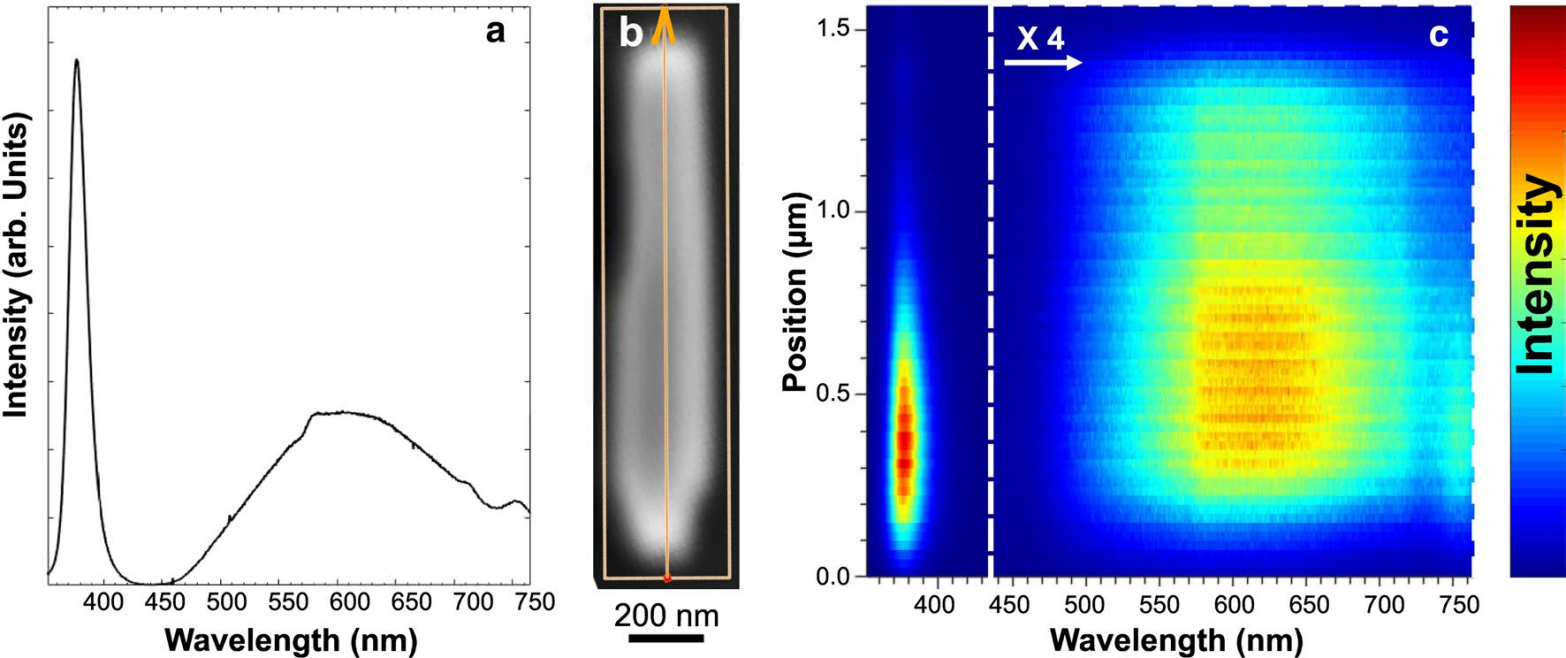
Cathodoluminescence data from a single ZnO NR transferred to a Si substrate. **a** A spectrum from the entire NR. **b** An SEM image of the investigated NR was recorded simultaneously with the luminescence data. The growth direction is along the arrow. **c** Hyperspectral luminescence map recorded along the line in **b**. The x-axis is the wavelength, the y-axis in the spatial position and the intensity is plotted in false color scale, as indicated in the figure. Note that the intensity of the defect band around 620 nm is enhanced by a factor of 4

Finally, in Fig. 7, we show spatially resolved cathodoluminescence mapping of a single ZnO NR. The cathodoluminescence study was performed in a dedicated SEM at room temperature, operating at 5 keV with a probe current of 25 pA. The luminescence was recorded by a CCD detector in hyperspectral mode, where a full spectrum is recorded in each pixel of the images. An average cathodoluminescence intensity spectra (Fig. 7a), and hyperspectral maps along a line (Fig. 7b), is presented as a false-color image in Fig. 7c. Interestingly, the strong near-band-edge emission, with a peak at 380 nm, was observed in the bottom segment of the NR. It becomes weaker further up, where instead strong deep-level recombination results in a broad emission band with a 620-nm peak position. The overall top view cathodoluminescence in Additional file 1: Figure S7, also shows the two spectral features luminescence in a larger area.

It is well known that the deep-level emission is due to the native point-defect levels of Zn and O vacancies in the crystal structure. The non-uniform spatial distribution of the deep-level emission thus indicates an inhomogeneous defect distribution along the NRs, with higher defect density in stronger emission regions. This local defect variation can be attributed to the change of the growth parameters, e.g., precursor concentrations, during the CBD process, as previously reported in the literature [34, 35].

As frequently reported in the literature, the vertical growth of ZnO NRs is claimed only by demonstrating top-view SEM images or integrated XRD diffraction patterns. But more accurate cross-sectional imaging typically shows that growth on non-patterned substrates, in the early phase, results in a compact, textured ZnO layer with vertical alignment at the top of the short NRs [36]. The individual NRs are more or less indistinguishable from one another, leaving no open paths to the substrate (Additional file 1: Figure S3a). In contrast, growth on patterned substrates results in NR arrays with open space in between. Evidently, the nanofabrication technique demonstrated here is capable of synthesizing a bottom-up, density-controlled, substrate-independent, and selective growth of single ZnO NRs with high quality. Because of the intrinsic behavior of epitaxial growth on a polycrystalline seed layer, the final NR arrays do not exhibit a perfect vertical alignment. However, a significant improvement in vertical alignment is readily observed compared to non-patterned samples (Additional file 1: Figure S3a). Further in-depth investigations are needed to further improve and control the critical crystal orientation of the seed layer.

## Conclusions

In summary, we realized an almost vertical growth of ZnO NRs on CL-pattered (100) Si substrates, precoated with a ZnO NP seed layer. The seed layer was uniformly deposited using a sol-gel technique by dip-coating the Si substrates in the sol-gel solution. Our findings show that two-step dip-coating enhances the smoothness and the crystal grain size of the seed layer, leading to a better NR alignment. Moreover, a selective area nanohole patterned resist template with tunable diameter and pitch was fabricated on the seeded substrates using CL. Subsequently, we grew a density-controlled array of single ZnO NRs in the patterned nanoholes by CBD and investigated them by XRD and cathodoluminescence with respect to crystal quality. Also, the ZnO NR growth stages were studied by SEM after different growth time intervals. Our demonstrated nanofabrication technique, offering simplicity, uniformity over large wafer-scale areas, and controllable growth of vertical ZnO NRs can be used to fabricate high-performance devices.

## Supplementary Information

**Additional file 1: Figure S1:** SEM images after different colloidal lithography steps. **Figure S2:** AFM image and surface line profile of dry-etched resist layer. **Figure S3:** SEM image of CBD-grown ZnO-NRs and vertical alignment distribution plots. **Figure S4:** Nanohole surface distributions on CL-patterned resist layers. **Figure S5:** SEM images of CBD-grown ZnO-NRs on CL-patterned ZnO-NP seed layers. **Figure S6:** EDS mapping of CBD-grown ZnO-NRs. **Figure S7:** Top-view cathodoluminescence mapping of ZnO-NR sample in Fig. S6.

## Data Availability

All data relevant for the reproduction of the results presented in this work are included in this published article or in its supplementary information (SI) file.

## Abbreviations

ZnO NRs: Zinc oxide nanorods
NPs: Nanoparticles
CBD: Chemical bath deposition
CL: Colloidal lithography
PS-NBs: Polystyrene nanobeads
DI: Deionized water
RIE: Reactive-ion etching
SEM: Scanning electron microscopy
EDS: Energy-dispersive X-ray spectroscopy
AFM: Atomic force microscopy
XRD: X-ray diffraction
RMS: Root mean square
RDF: Radial distribution function
