# Supplementary material for: Synthesis of Vertically Aligned ZnO Nanorods Using Sol-gel Seeding and Colloidal Lithography Patterning
Source: Nanoscale Res Lett. 2021 Mar 12;16:46. doi: 10.1186/s11671-021-03500-7 (PMC7952483; doi:10.1186/s11671-021-03500-7)
Supplement: Supplementary file 1 — Additional file 1: Figure S1: SEM images after different colloidal lithography steps. Figure S2: AFM image and surface line profile of dry-etched resist layer. Figure S3: SEM image of CBD-grown ZnO-NRs and vertical alignment distribution plots. Figure S4: Nanohole surface distributions on CL-patterned resist layers. Figure S5: SEM images of CBD-grown ZnO-NRs on CL-patterned ZnO-NP seed layers. Figure S6: EDS mapping of CBD-grown ZnO-NRs. Figure S7: Top-view cathodoluminescence mapping of ZnO-NR sample in Fig. S6. [file 11671_2021_3500_MOESM1_ESM.pdf]

Supplementary information

Synthesis of vertically aligned ZnO nanorods using sol-gel seeding and colloidal lithography patterning

Ebrahim Chalangar [1,2], Omer Nur [1], Magnus Willander [1], Anders Gustafsson[3] and Håkan Pettersson [1,2,3*]

[1] Department of Science and Technology, Physics, Electronics and Mathematics, Linköping University, Norrköping, Sweden
[2] School of Information Technology, Halmstad University, SE-301 18 Halmstad, Sweden
[3] Solid State Physics and NanoLund, Lund University, Box 118, SE-221 00 Lund, Sweden
* Correspondence: hakan.pettersson@hh.se; Tel.: +46-35-16-7306

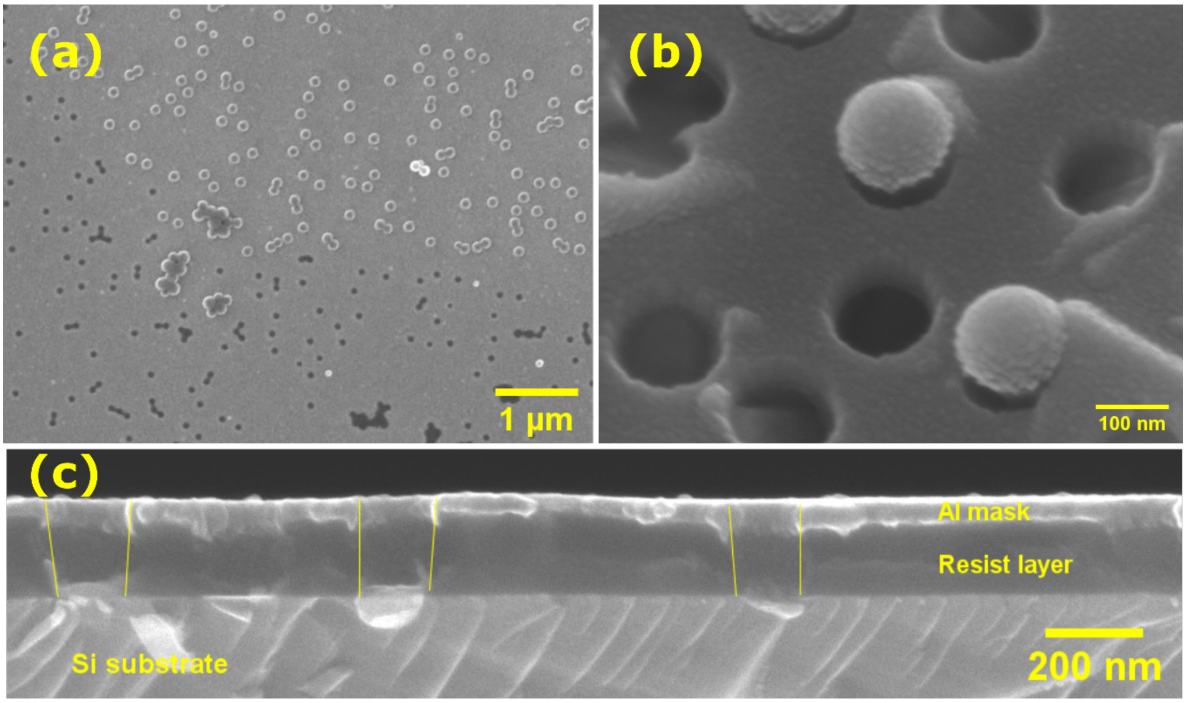

Figure S1. SEM images after different colloidal lithography steps. (a) Partially removed Al-coated PS-NBs by tape stripping. (b) Tilted (5°) image of the tape-stripped surface in (a) at higher magnification. (c) Cross-sectional image of the resist layer after dry etching.

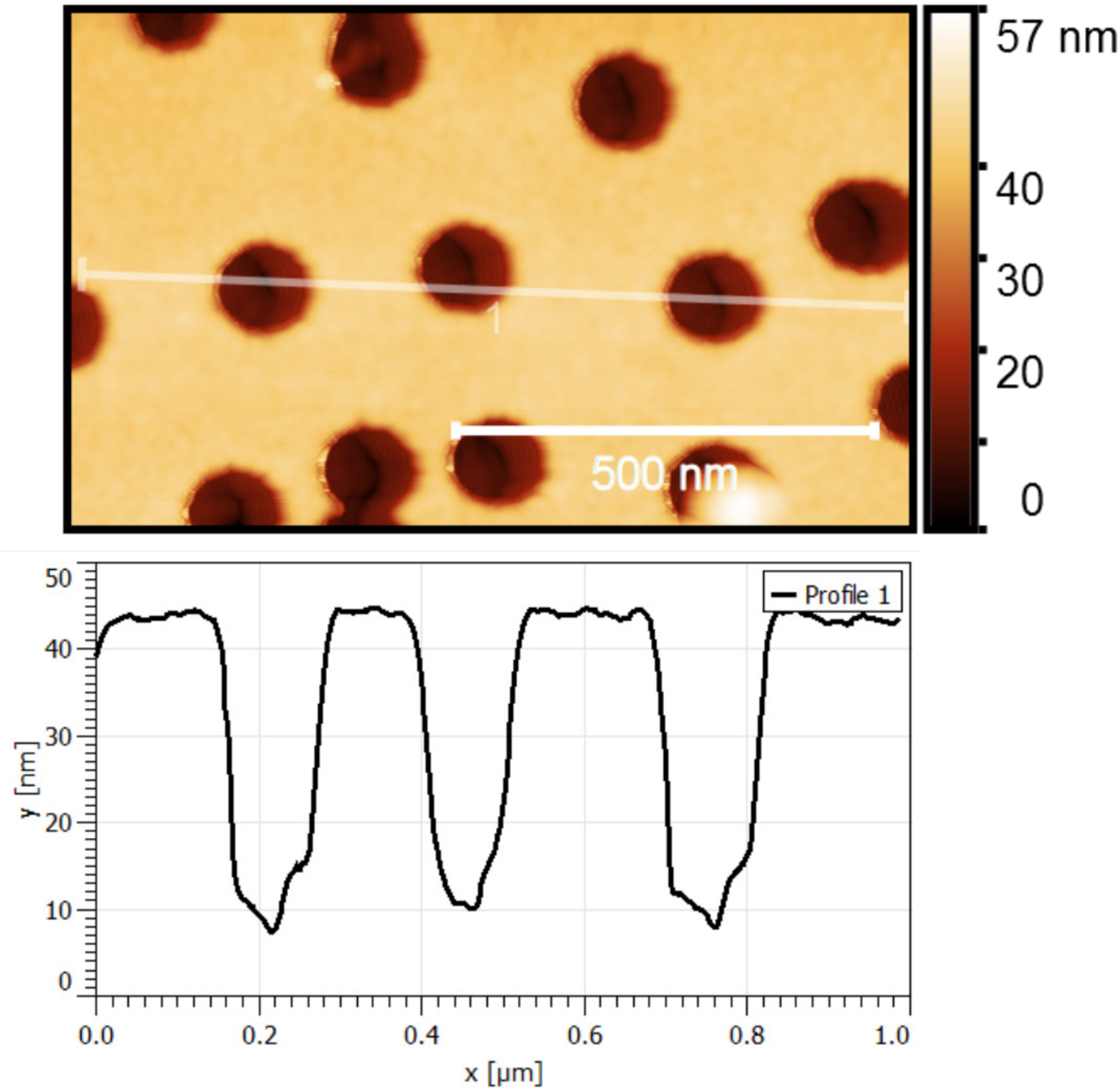

Figure S2. AFM image of the dry-etched resist layer on top of a Si substrate (top), and a surface line profile of the sample (bottom).

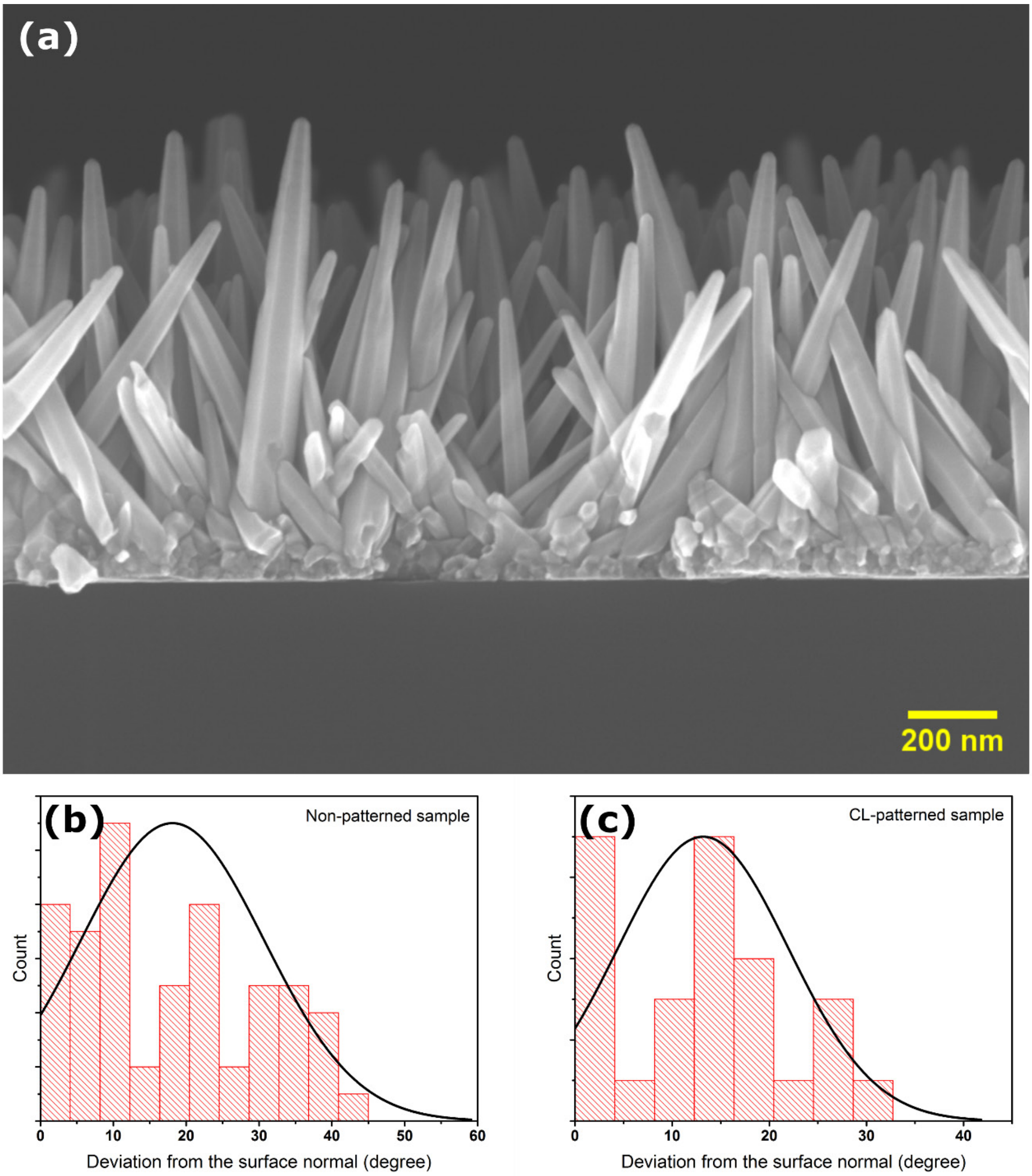

Figure S3. (a) Cross-sectional SEM image of CBD-grown ZnO-NRs on a non-patterned ZnO-NP seed layer. Distribution of the deviation angle from the surface normal for (b) a non-patterned sample, calculated from the SEM image in (a), displaying a mean deviation angle of 18°, and (c) for a CL-patterned sample, calculated from Figure 5c, displaying a mean deviation angle of 13°.

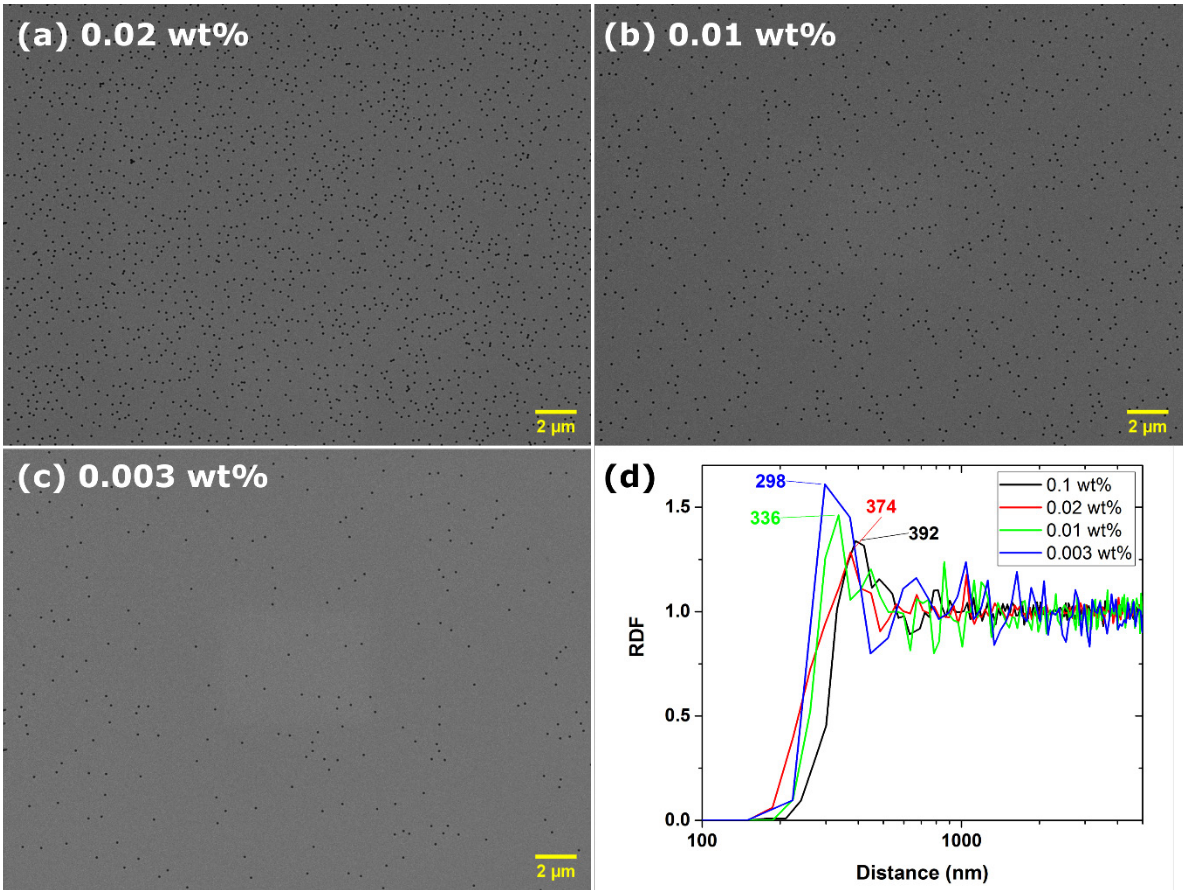

Figure S4. Nanohole surface distributions on CL-patterned resist layers on (100) Si substrates. The CL was done using 140 nm diameter PS-NBs with different suspension concentrations of (a) 0.02 wt%, (b) 0.01 wt% and (c) 0.003 wt%, resulting in nanohole surface densities of 3.2, 1.5 and 0.4 nanoholes/μm^2, respectively. (d) The corresponding RDF of the patterned nanoholes for different PS-NB concentrations display average separations of 374, 336 and 298 nm, respectively. The black trace shows the RDF for the 0.1 wt% suspension, used for the sample in Figure 4a, exhibiting an average nanohole separation of 392 nm.

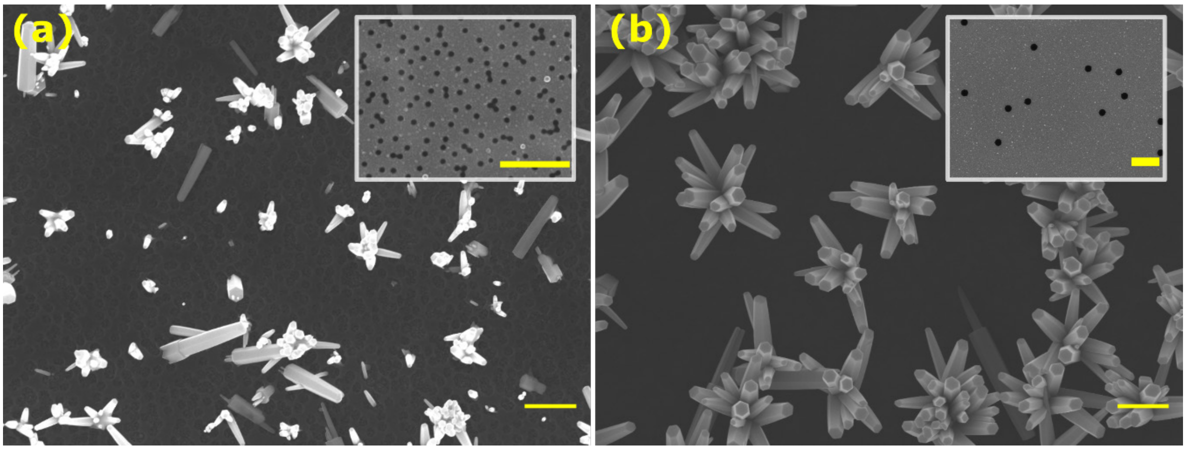

Figure S5. SEM images of CBD-grown ZnO-NRs on CL-patterned ZnO-NP seed layers on (100) Si substrates using (a) 107 nm and (b) 320 nm diameter PS-NBs. The insets show corresponding CL-patterned resist layers after tape stripping of the Al-coated PS-NBs. The scale bars are 1 μm.

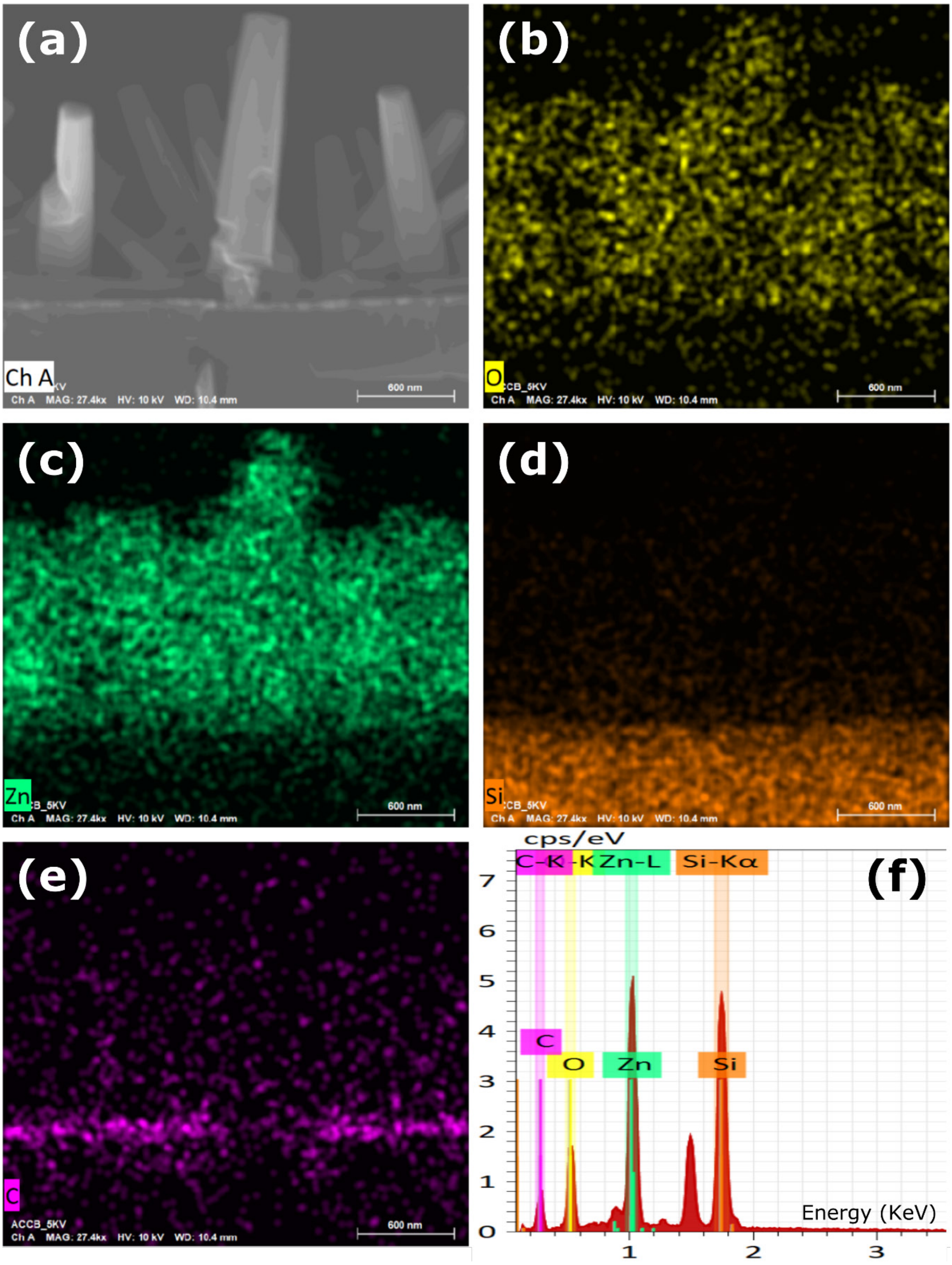

Figure S6. (a) Cross-sectional SEM image and spatial EDS chemical mapping of (b) O, (c) Zn, (d) Si and (e) C for CBD-grown ZnO-NRs on CL-patterned ZnO-NP seed layers on (100) Si substrates. (f) EDS spectra of the different elements. The scale bars are 600 nm.

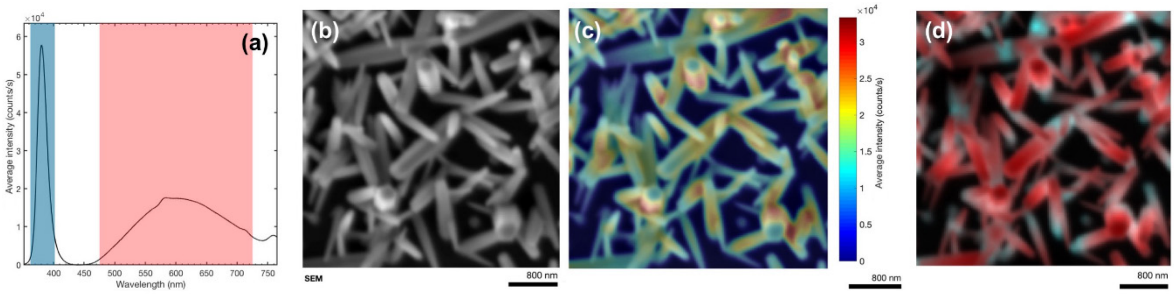

Figure S7. Top-view cathodoluminescence data from many as-grown ZnO nanorods. (a) An average spectrum collected over the sample area, shown in (b). (b) A typical SEM image of the sample. (c) Color-coded luminescence from the entire spectral range overlaid on the SEM image in (b). (d) A color composite image of the near bandgap emission (blue) and the defect band (red - enhanced by a factor of 3).
